# “(Meth) Will Hurt You and Hurt Your Teeth”: Teen, Parent, and Dental Practitioner Perspectives on Implementing Crystal Meth Use Prevention Messaging in the Dental Office Setting

**DOI:** 10.1155/2022/6933091

**Published:** 2022-05-04

**Authors:** Margie R. Skeer, David M. Landy, Emma C. Ryan, Michelle Lee-Bravatti, Tamar Boyadjian, Jennifer Towers

**Affiliations:** ^1^Tufts University School of Medicine, Boston, MA 02111, USA; ^2^Gonzaga University, Spokane, WA 99258, USA; ^3^Tufts University School of Dental Medicine, Boston, MA 02111, USA

## Abstract

**Objectives:**

Crystal methamphetamine (“meth”) use among youth living in rural areas is higher than the national average. Given how drastically meth affects teeth (i.e., “meth mouth”), engaging dental professionals as one of multiple channels in rural areas to deliver meth prevention messaging is a novel approach. The objective of this research was to assess the feasibility and acceptability of incorporating meth use prevention messaging into dental visits with teenagers.

**Methods:**

We conducted phenomenological, qualitative research with dental practitioners, teens, and parents/guardians in three communities in North Idaho, from 2015 to 2016. We recruited practitioners using a snowball sampling strategy and placed phone calls to dental practices and contacted teens and parents through schools, libraries, local sporting events, and word-of-mouth. Using NVivo 12-Plus, parent- and teen-specific codebooks and themes were developed from guides and transcripts. Transcripts of the dentists and hygienists were reviewed to ascertain the main ideas and themes.

**Results:**

Overall, practitioner, teen, and parent participants viewed meth prevention messages delivered by dental professionals as acceptable and feasible. Compared to those in private practice, public health dental providers were invested in meth prevention and were eager to help. Barriers to overall acceptability and feasibility included hygienists' low self-efficacy to deliver a communication-based intervention, infrequency of dental visits impacting the ability to reach enough teens through this venue, and the fact that teens could feel “targeted” by providers. Teens also raised concerns about scary messages exacerbating preexisting dental visit anxiety. Facilitators included the following: dental practitioners already engaging in health education with their patients, parents, and teens seeing dental professionals as appropriate purveyors of antimeth messaging and support for increased meth prevention efforts given the impact of meth use in their communities.

**Conclusions:**

Well-crafted, developmentally appropriate meth prevention messages would likely be well received by teens and supported by parents in dental offices. These data are being used to develop a novel, theory-based communication and behavioral strategy to integrate dental professionals into the delivery of messages aimed at preventing the initiation of meth use among rural Idaho teens.

## 1. Introduction

Frequent and regular use of crystal methamphetamine (“meth”) via smoking, injecting, snorting, or ingesting can cause extreme deterioration of the mouth and skin [1]. Considered a growing epidemic in the dental field [2], “meth mouth” is the result of an array of dental diseases caused by meth use, including dental caries, breakage, cracking, deep discoloring, rotting [3, 4], and ultimately intense oral pain [5]. In one study of 571 people who use meth, 96% had caries, 58% had untreated dental decay, and 67% did not have all of their natural teeth [3].

Meth profoundly affects the teeth for many reasons. First, the drug mechanism reduces saliva production, leading to chronic dry mouth (xerostomia) and subsequently increased consumption of sugary beverages (three times greater than those who do not use) [6, 7]. Second, meth use is associated with teeth grinding and clenching (bruxism), which results in significant wear on the teeth [8]. Third, drug addiction can lead to reduced oral hygiene practices, such as not brushing the teeth, snacking, smoking cigarettes, and not regularly attending dental visits [7]. Combined, these issues are a significant catalyst for the development of dental decay.

Given the devastating effects of meth use disorders [9], there is a need for innovative meth use prevention strategies targeted toward youth, particularly in rural areas, where meth use poses a significant risk for adolescents [10]. Although meth use is a national concern, there is a distinct impact on rural communities, with those in rural areas having a lower age of initiation and a higher burden of psychiatric comorbidities as compared to those in more urban areas [11]. Law enforcement officers in Idaho and Oregon stated in a 2019 survey that meth is the “most prevalent illicit drug” and “greatest drug threat” [12], which is consistent with other findings showing that three-quarters of law enforcement agencies in the Pacific and Western regions of the country report that meth is the most significant threat facing these areas [13]. The impact on youth specifically is of concern, as past year use among 12th grade students increased over twofold in a five-year period, from 0.6% in 2015 to 1.4% in 2020 [14, 15]. Due to the lower age of initiation of meth use in rural areas (e.g., 2.4% in 9th grade compared to 1.4% in 12th grade) [16], it is important to develop prevention messaging strategies that reach individuals in these areas at a younger age. A former state program in Idaho aimed at school-aged children, The Idaho Meth Project, was a nonprofit organization based on The Meth Project model that started through a philanthropic gift. Though now discontinued, the organization's classroom outreach, both in person and online, in part contributed to Idaho's approximate 50% decline in meth use during its existence [17]. The organization and its programming ceased to exist circa 2018 due to a lack of funding, thus creating a significant need for health messengers dedicated to meth use prevention.

Individuals delivering public health messages are considered effective and persuasive if they are perceived to be objective and credible [18, 19]. Dental practitioners in particular have been identified as “enablers of change” in delivering preventive care, with experience educating patients around sensitive public health topics [20–26]. The relatively long durations of dental appointments—compared with those in a managed, primary care health setting—provide an important opportunity for the communication of health promotion and risk prevention messages. Research conducted previously with dental hygienists in rural North Idaho indicated that they spend a significant amount of every visit educating patients about health topics such as human papillomavirus, disordered eating, oral cancer, gastroesophageal reflux disease, malnutrition, and tobacco use, both due to their oral effects and in an effort to provide comprehensive patient care [27]. While dental visits offer worthwhile opportunities to build upon existing health education with the addition of meth use prevention initiatives, a tailored, interpersonal, evidence-based approach in the context of a dental setting should be tested and explored [28]. To this end, we can look to theoretical frameworks and conceptual models used in other areas of health education and research to inform meth use prevention efforts.

The Social Cognitive Theory (SCT) [29] lends a roadmap for understanding behaviors in this context. The SCT asserts that an individual's combined built and existing environments interact in a reciprocal manner with their personal characteristics, a concept known as reciprocal determinism [30]. Because this theory explains how determinants at multiple levels (i.e., individual, interpersonal, societal, and environmental) of the social-ecological model are interwoven, it can be useful to apply to complex health behaviors such as substance use, where such determinants can play equally important roles. This can also be explained by the construct of observational learning, whereby teens learn from the behaviors of those within their social contexts, which tends to shift from caregivers in childhood to peers in adolescence [31]. Skills building in the realm of self-efficacy (a key construct of the SCT) [29] to refuse offers is critical, as self-efficacy to resist initiation and refuse offers of substances has consistently been shown to be a strong predictor of reduced adolescent substance use [32–35]. Finally, self-regulation, the way teens interact with environmental incentives as they relate to consequences, applies to the decisions they make with respect to choosing and spending time with people they know to be using meth—or at least do not perceive its use unfavorably [36]. To the best of our knowledge, this approach toward meth use prevention has never been developed nor implemented in a dental setting.

The objective of this qualitative study was to examine the acceptability and feasibility (e.g., suitability of the setting and messengers for effective operational delivery) of incorporating a health communication messaging-based meth use prevention intervention into the dental setting in North Idaho. These were assessed from the perspective of dental practitioners as the target for the intervention delivery, teens as the recipients of the intervention, and parents/guardians (referred to as “parents” herein) as those who may need to provide permission for intervention delivery. We aimed to explore whether or not it was realistic for dental hygienists to engage in this type of prevention work from their perspectives and from those of their employers (i.e., dentists) and if a meth prevention program would be well received and supported by the teens, the intended audience, and their parents.

## 2. Methods

The epistemological approach to this area of study is constructivist in nature as outcomes are dependent on socially constructed attitudes and perspectives of individuals and the interplay between them. Using an interpretivist theoretical perspective [37], we employed a phenomenological methodology to gather data about the lived experiences of dental practitioners and teenage patients and parents.

The interviews and groups were conducted by a behavioral and prevention scientist who designs substance use preventive interventions for adolescents (MS) and a research administrator in the field of dental medicine who also has a health communication background (JT). This investigative team had a significant understanding of behavior change and substance use prevention theories, the dental setting, and qualitative research. Prior to conducting the research, the investigators worked closely to understand the dental providers, teens, and parents and address preconceived notions about the population.

Between May 2015 and September 2016, we conducted key informant interviews (KII) with dental practitioners (dentists and dental hygienists), followed by focus groups and small group interviews with teens and parents of similarly aged adolescents. We conducted individual interviews with providers because we sought to understand their unique perspectives on incorporating a prevention program into their specific practices, therefore making in-depth interviews more relevant [38]. For the teens and parents, we aimed to elicit feedback through the context of a discussion to spark new thoughts and ideas, making focus groups the best strategy [38].

Groups for teens and parents were offered over a range of days and hours, yet many eligible participants expressed not being available or not wanting to engage in a discussion about meth use or prevention due to the personal nature of the topic for many residing in the region. Therefore, while we initially planned to conduct focus groups, we recruited smaller numbers for each group session. As a result, we conducted both focus groups (with six or more participants) and small group interviews (with fewer than six participants). The protocols for this work were reviewed and approved by the Tufts University Health Sciences Institutional Review Board (approval numbers 10787 and 10808).

### 2.1. Key Informant Interviews

Dental practitioners were recruited using snowball sampling through emails and phone calls to dental practices in the Idaho Panhandle using an approved recruitment script for both phone and e-mail. Inclusion criteria were being over 21, having been licensed and practicing for at least three years, speaking English as their primary language, and, for dental hygienists, working in a practice that employed at least two people in their role. We included English-speaking as one determining factor for inclusion because the study team did not have the skills to conduct interviews/focus groups in other languages and did not have the resources to hire interpreters. We opted to have a minimum age of 21 and a minimum experiential threshold of three years to account for a level of professional experience to answer questions about incorporating meth prevention into the visit. Furthermore, dental practices with less than two dental hygienists would make it more challenging to consider any additions to their scope of practice, including meth use prevention work. We targeted dentists who owned their own practice, but this was not a criterion for those working in dental public health clinics.

### 2.2. Focus Group and Small Group Interviews

We recruited teen and parent participants through a convenience sampling approach via social media, in-person recruitment at community events, and flyers posted at schools, dental clinics, and other community spaces throughout the Idaho Panhandle. Eligible teens (1) were between the ages of 12 and 19, (2) spoke English as their primary language, (3) received preventive dental care with a dental hygienist at least once per year on average, and (4) had obtained written permission from a parent/guardian to participate in research (for subjects aged 12–17). Parents were eligible to participate if they had at least one child aged 12–17 who received preventive dental care with a dental hygienist at least once per year on average.

### 2.3. Data Collection

KIIs, focus groups, and small group interviews were conducted by the primary investigators (MS and JT) using theoretically relevant, semistructured interview guides, were administered in English, and ran approximately one hour long. We summarized key findings at the end of each group. Groups were audio-recorded to be transcribed and, following the completion of each interview/group, transcriptions were completed and checked for accuracy. Informed consent was obtained for participants over 18 and parental consent was obtained for those under 18, with an additional information sheet for the latter group. Dental practitioners were compensated $35 for completing the interview and teens and parents were compensated $20 for participating in the groups. For KIIs with hygienists and dentists/practice owners, general topics grounded in SCT were selected to explore both groups' feelings of self-efficacy with respect to practice-based meth use prevention interventions, the perceived barriers and facilitators to adopting new behavior within their clinical practice(s), the participants' notions about their teen population's susceptibility to meth use, their feelings about the severity of meth use in their populations (both local and practice-based), the acceptability and feasibility of incorporating meth use prevention interventions into their practices, and the likelihood of doing so if given the opportunity.

Guides for teens were informed by existing meth prevention messaging research [39], the SCT, and the Extended Parallel Process Model (EPPM) [40] and were devised to explore their awareness and understanding of meth use and addiction, their opinions about the effectiveness of fear-based concepts and images that have been used in another meth use prevention campaign [41], and their preferences for future content, as well their feelings about the acceptability and feasibility of fear appeal messaging within a dental setting. To better understand this, we showed teen participants a set of media and clinical images [28]. To communicate the effect of displaying physical changes as a result of prolonged meth use, we showed police arrest photographs taken of individuals before or early in their meth use and after sustained use. We ended by discussing the concept of a program that would simulate teeth and skin changes over time as a result of meth use. The guides are available upon request to the corresponding author.

For the parent groups, guides were written to explore their perceptions of their own conversations about substance use with their children, their feelings about their children's susceptibility toward meth use and its severity in their community, their awareness of meth's physiological effects on the skin and teeth, and their attitudes toward acceptability and feasibility of meth use prevention programming in a dental office setting including illustrating physiological changes to the mouth and skin to teens.

For all stakeholder/audience groups, we rooted our questions in topics related to the feasibility and acceptability of implementing a meth use prevention campaign targeting adolescent patients within a dental office. We also included questions to assess the likelihood of behavior change among the different groups as a result of the intervention. More specifically, for dental practitioners, the behavior change would be adopting (for hygienists) and endorsing (for dentists/practice owners) new educational and communication initiatives related to meth use prevention. For teens, it would be the likelihood of refusing offers of and resisting meth use, and, for parents, the likelihood of supporting/endorsing the dental practice-based intervention.

### 2.4. Analysis

We transcribed the interviews and groups verbatim and cross-checked each transcript for clarity and accuracy. For the teen and parent groups, we employed a deductive qualitative content analysis method [42, 43] where we created two provisional code lists (one each for teens and adults) based on the interview guides. Revised sets of codes were developed as additional questions arose during the course of the initial teen and adult groups. Once the code sets were revised, each researcher worked to independently code a transcript to ensure its accuracy and completeness. Transcripts were reviewed, and each set of codes was updated as needed to include new items and redefine existing codes in order to develop the final codebooks. The research team reviewed all changes and worked through any disagreements to arrive at a set of final codebooks and definitions that everyone agreed on. Saturation occurred when no additional codes emerged [44]. To ensure interrater reliability, we used the NVivo qualitative data analysis software tool (V12 Plus) to apply the final coding frameworks to each transcript (average Cohen's Kappa score: 0.67). Three members of the research team coded four of the five transcripts, and one member coded the fifth transcript once an acceptable level of interrate reliability was established. Once coding was complete, we analyzed the coded transcripts to identify key themes related to health communication strategies and messaging techniques. We reviewed the transcripts of the dentists and hygienists independently to ascertain insights from the dental practitioners in the context of findings from teen and parent themes.

## 3. Results

We conducted KIIs with six dentists (all male) and six dental hygienists (all female) from nine practices and subsequently small group interviews and focus groups with teens (*n* = 3) and parents (*n* = 2). Groups for teens and parents were offered over a range of days and hours, yet many eligible participants expressed not being available. As a result, we conducted both focus groups with six or more participants and small group interviews with fewer than six participants. Two teen groups were with middle-school-age students and one was with high-school-age youth. Middle-school-age and high-school-age participants were separated to maintain developmental appropriateness. Teen and parent participants were all white and resided in rural North Idaho. While not a criterion for inclusion, approximately half of parent participants were related to teen participants. Though we did not screen for participant knowledge or experience with addiction and/or meth use, our sampling approach resulted in groups where most of the participants had witnessed or experienced addiction and/or meth use first-hand.

In general, participants understood meth use and its prevalence in North Idaho, which made the discussion about prevention measures most relevant. Dental practitioners were highly aware of crystal meth as a problem in the area and of the consequences of use and addiction.

“When you get to Idaho, you see it pretty rampant. And so I can identify with a lot of patients (who) are really straightforward. You see them and they are like, okay, I'm trying to get my life back together. My mouth is completely destroyed; this is just one more step in rehab.” (Hygienist: private practice)

Teens and parents were also acutely aware of the prevalence and impact of crystal meth use in their communities. As described previously [28], most in the sample personally knew people who experienced addiction to meth and all understood the oral and physical consequences of meth use.

In the current study, two primary themes were identified from the groups with teens and parents, which were corroborated with the KIIs: (1) considerations for implementation in a dental setting and (2) innovative strategies and context. Here, we organize the results first by sentiments about meth prevention messaging being delivered by dental practitioners, next by barriers and facilitators to acceptability, and finally by barriers and facilitators to feasibility.

### 3.1. Sentiments about Implementing a Meth Prevention Intervention in a Dental Setting

Overall, clinicians, parents, and teens generally found the concept of meth prevention messaging in the dental setting acceptable, with some caveats. While some felt that messaging should be tailored for age as it may “*freak kids out”* if they are too young, many thought it was a good idea outright.

“Yeah, it'd have to be like a conversation just leading into like hey this can happen… meth mouth. But I feel like it could be really valuable because (dentists are) really smart…” (Middle-school-age teen)

In general, parents wanted their kids to get information and prevention messaging about drug use (*“I think information is key—you have to stay informed”*) and felt that the dental clinic would be an appropriate place to educate teens about the dangers of meth use. They saw the connection to the oral cavity and thought it was a logical venue for this type of education.

“I think that would be perfect, seeing as how ‘meth mouth…' You're sitting there working on them and you can, you know, talk to them about it, about you know, this will hurt you and hurt your teeth. I think it would be perfect.” (Parent)

“I mean, yeah, if they're talking to my kids about making sure you brush twice a day or something like that, I don't see any problem with them talking about don't use drugs. I think that anywhere they can get that is going to be good.” (Parent)

Dentists in particular had varied experiences with respect to meth mouth in the dental clinic. Generally, dentists who owned their practice or were in practices focused on cosmetic or pediatric dentistry did not see meth-related dental problems and were, therefore, less amenable to the idea of incorporating messaging around meth use prevention into the dental appointments.

“I was just thinking… what would I want to come my direction if I was a parent? I don't want to see this here and I don't want to walk in and see a picture of someone with meth mouth, no I don't… You have to be a little delicate with how it's presented.” (Dentist: Private, cosmetic practice)

But dentists working in dental public health clinics who saw the effects of meth use, particularly among parents, found the concepts acceptable and were eager to help.

“Public health providers are probably a good place to start because… there's a reason why we do what we do.” (Dentist: Dental public health clinic)

“People say there's meth all around here… it's becoming more of an issue…. I would be more than happy to help in whatever I can do to move (meth prevention work) along to help our community be better. If we can start education… at a younger age and kind-of stop these trends that are happening, that will help, hopefully.” (Dental hygienist: Dental public health clinic)

### 3.2. Barriers and Facilitators: Acceptability

Major barriers to acceptance and efficacy of the preventive intervention raised by teens and parents included invoking fear, feeling targeted by the provider, and the potential to incite curiosity about meth. Some felt that the dentist's office was not an ideal venue for this messaging for reasons ranging from dislike or fear of the dentist to them/their parents not wanting to spend more time there.

“I don't know because a lot of people are already really afraid of the dentist and so I think being afraid of the dentist and going there and then talking about meth would probably not be a very good experience.” (Middle-school-age teen)

“I don't think it would be the best to do it at the dentist's because parents don't like waiting for hours or you know more than like thirty minutes for the kid to get done at the dentist. Most parents do not who does… you're just sitting there. Plus, like you already have to worry about cavities crap you need done and probably wouldn't want to talk about what could happen.” (Middle-school-age teen)

Teens and parents raised another barrier; that is, young people are often scared of the dentist, so talking with them about meth could make the experience even worse.

“I think it would be a great source… I think you almost have to hear this message from different sources, but definitely I think it's one area. And they very much like Dr. ____ so I think they would listen, but at the same time they're traumatized, they're sitting there stressing over what's being done and things like that, so depending on how it was delivered.” (Parent)

Regarding facilitators, dental practitioners discussed talking with teenage patients about issues that affect their teeth other than brushing, such as smoking or using dip, so discussing meth would be aligned with what they are already doing.

‘I think it's a great avenue to give the information out especially to those teens that are right in that age…' (Dental hygienist: Private practice)

In asking parent respondents about facilitators to acceptance, while parents were generally amenable to the idea of prevention messaging being delivered by dental practitioners, parental education and consent were raised as a big issue. The parents did not explicitly say that they needed to be educated prior to their children receiving prevention programming but expressed that a quick explanation surrounding the goals for that visit (with a focus on prevention) would go a long way with the parents. They wanted to know what the hygienists were discussing with their children, particularly if what they were discussing was negative. This was suggested as a way for parents to not feel that the clinician was accusing their child of using meth; rather it is something they should do with all teenage patients.

‘… I think it just boils down to respect for parents and their rights, especially with the big Parental Rights Movement in the state. That you just communicate with them… here's what we're going to discuss this semester, here's what we're going to present to your child… You know, here you go to the dentist and you say you know they're at that age where we're, you know, we wanna just kind of focus on some prevention and share some things that we, could happen with your oral health if you made some of these choices…' (Parent)

‘(Permission would be) like, look, I've been asked to talk about this, so, kind of little introduction, this is not me trying to avenge you or accuse you of anything. I've been asked to talk to you about this.' (Parent)

Some dentists and hygienists agreed with the need for parental permission before talking with teens about meth use—even prevention messages as well.

‘I think you'll need a little bit more permission because that's kind of personal, maybe my child might be more sheltered and they may feel they don't need to. I think we need to get permission.' (Hygienist, private practice)

Some parents felt that educating them prior to talking with the teens would be important so that parents could reinforce messaging and answer questions. It was also raised that the message should be framed strictly through a dental lens so that the practitioner can maintain credibility—straying too far from why the patient is at the visit could threaten the credibility and patient comfort level.

‘It boils down to relationship and communication with your patients but I would not have a problem if they had said to him—he's 14—okay, we're going to go over some pictures today about dental health and the effect drug use has on your dental health. And the effects that chew has on your dental health, smoking, meth, all of those things. And I guess at his age, I would say thank you.' (Parent)

However, practitioners in public health clinics did not have this concern because many of their teenage patients came to the clinics without parents and, since many had parents who were addicted to meth or were incarcerated for crimes related to meth use, they felt comfortable discussing this topic with teens in their practices.

‘(Parents) are hardly ever present, a lot of them won't even come in, they just drop them off. So a lot of the teenagers just come by themselves…, there is not a lot of parental guidance and involvements and usually if there is, it's usually the people who are not in that category [referring to those at higher risk for meth use].' (Dentist, Public health clinic)

Another facilitator to acceptance was that, as previously mentioned, teens felt that an application showing simulated effects on their own appearance after a duration of meth use would be an effective strategy (*“You'd have to make it look skinner, eyes sunken in, crank bugs…”; High-school-age teen*). In exploring this further, there was a strong consensus that if personalized and realistic, this type of technology would be accepted positively *(“I think definitely something like that would work”; Parent)*; however, the image processing program would not be useful if it was perceived as *“just another picture app” (High-school-age teen).* Parents were especially supportive upon learning that such technology would only be used in the context of the dental visit (i.e., the program would not be publicly available to download) (*“Coming from a doctor, that may make it a little bit more serious”; Parent)*.

### 3.3. Barriers and Facilitators: Feasibility

From the clinician's perspective, major barriers included concerns about the appropriateness of content and self-efficacy to convey such messages. While almost all hygienists felt that messaging around meth use and “meth mouth” would be in line with the type of prevention messaging they deliver to teens, they would need to be careful to not be too intrusive, as it can be a very private and complex matter.

‘It's a fine line of where I don't want to intrude on their privacy but right out of the back, I can usually tell if they are chewing or smoking and I will ask them about it. And some of them would say no and you'd know they are lying because the smell of the smoke on their breath. You'd see the chew...the residual effects of the chews. And they will say no and so, then I would just say... let them know what they could do, you know.' (Dental hygienist: Private Practice)

Furthermore, hygienists expressed a lack of self-efficacy in delivering meth-specific content to teens and a need for further training on the topic.

‘Personally, I would like to be better educated on it. I mean, I don't feel like I am as educated on it as I should be.' (Dental hygienist: Private practice)

‘I would like to know more about the early signs not when it's full blown. Is there anything that is um, are there any signs that we should start looking for before they start having physical effects?' (Dental hygienist: Private practice)

Some teen participants were concerned that a barrier to providing messaging about meth in dental practices could be that they do not go to the dentist often—at best, two times per year—or some cannot afford the dentist at all, so this type of message may not have enough of a dose to be hard-hitting.

‘Because like some kids go to the dentist but it's like every six months no one's going to really pay attention to it when they're in the dentist's office it's like get it in get out yeah but if you see it every single day in school.' (High-school-age teen)

‘That's where the breakdown is you don't have kids that are fortunate enough to have the kind of dental care that, you know, we provide our kids.' (Parent)

Another concern raised by teens, which was echoed in one of the parent groups, was that if the dentist or hygienist brought up meth use during the visit, the patient might start to wonder if the dentist assumed that they were taking drugs (*“Like do you think am tweaking?” High-school-age teen*). Furthermore, several of the teens were worried that if you raised the topic of meth with teens, they could get curious and want to try it.

“There are some kids out there that are so F-ed up in the brain already maybe from parents or something that they're going to be like. I think I'm invincible and it may not turn out like that so let's go and try it let's go in for it and I don't think that's going to hit me like that…” (High-school-age teen)

One parent raised an interesting concern that teens could use it as a way to plan how long they should use meth for a certain purpose, rather than it being a deterrent.

“It would be interesting to say,… here's 2 months, 4 months, 6 months. But then again they might say well I'm not gonna do this for eight months, I just wanna lose weight for my prom. Because that's another thing, that's where my stepdaughter started, as a weight loss thing.” (Parent)

With respect to facilitators, providers discussed having visuals and handouts for issues that affect the teeth (*“I do have some visual aids that I would pull out and show them as far as chew,” Dental hygienist: Private practice)*, so this would be aligned with practices they already implement. Sentiments on parent buy-in and permission were also raised by private practice dental practitioners; they wanted materials to share with parents to increase their acceptance.

Parents also raised the ways dental clinicians communicate with families, including providing relevant materials as a facilitator to this type of intervention (“*… even like a pamphlet that's sent home with the kids, I would be fine with something like that. As a ‘here's what we talked to your child about today'.”; Parent*). They agreed that education about meth prevention should start young—much younger than 13 years of age (*“I think it should be young.” “Yeah, the sooner, the better”*; *Parent)*. This was reiterated by the teens, who said that young people start learning about meth in middle school, mainly from their peer groups. However, teens also said that messaging should be developmentally appropriate so that young people can understand it and not be too young as to unduly scare them.

“I think that they should maybe start introducing it to like sixth grades and stuff because like when you're in seventh and eighth grade, like that's usually when you're start kind of experimenting -sometimes more towards freshman year, but just like depending on the group you hang out with so sometimes in seventh and eighth grade, it's a little too late. And that's usually when you learn about it.” (Middle-school-age teen)

“I think it would depend on their like the maturity level. If it was a little kid like 3^rd^ grade that you showed that, they'd be scared to death, but maybe like a couple years and they saw and heard it around, you know they would not be as much.” (Middle-school-age teen)

## 4. Discussion

We conducted phenomenological, qualitative research to explore the acceptability and feasibility of a new and innovative prevention strategy of delivering meth prevention messaging in the dental setting. Although all interviewed dental practitioners were aware of crystal meth use as a problem, those in public clinics found the intervention to be more acceptable, appropriate, and necessary to their practices than cosmetic and pediatric dentists, who expressed that they do not tend to see meth-related dental problems in their practice or perceive a prevalent threat of meth use among their patient bases. Given meth use in North Idaho and the risk of initiation to teens, these disparate sentiments among dental practice owners (segregated by practice type) were not expected—especially because we did not pose questions related to practice type specifically—but were an important finding that emerged from the analysis. There could be many reasons for these discrepancies and disparate experiences including the type of practice (e.g., specialty practices versus qualified public health clinics) and characteristics of the patient populations at the different practices (e.g., socioeconomic status, social determinants of health, age, etc.) that may increase a person's risk for initiating meth use. Upon further reflection, the sampling model through professional networks likely catalyzed the inclusion of varying types of dental clinics since the public health dentist participants were referred to us by specialty (e.g., pediatric/cosmetic) practitioners who were interviewed early in the study because of their specific patient populations and perceived relevance of our work. Given the opinions among participants that public health clinics would be an acceptable setting for a dental health communication effort related to meth use and prevention, it may be more feasible to implement the intervention primarily in dental public health clinics. While that may reduce the reach of the intervention, it may allow for more targeted messaging for those at higher risk, aligning with a “selective” prevention approach [45].

Dental hygienists were enthusiastic about the intervention but felt that they would need training in order to increase their knowledge, comfort level, and skills around meth prevention messaging to be able to successfully deliver the intervention. Given that hygienists already deliver public health prevention messaging in the dental setting, training them to deliver messaging around meth would build upon current skill sets. Additionally, the existing practice of using digital dental photography [46] and self-change imagery in dental settings and in a variety of public health interventions [47–49] may make the implementation of a meth prevention communication strategy more feasible and acceptable.

Dental hygienists are trained to clean and assess patients' teeth for current and future oral health issues and often use a strictly educational style when providing clinical advice [50]. In behavioral sciences generally as well as in the dental field, it is well established that providing patients information alone or warning them of severe ramifications is not enough to elicit behavior change [51–53]. Therefore, including a brief motivational interviewing- (MI-) informed component will be critical to increasing behavioral uptake. MI training has been developed specifically for dental hygienists [54, 55] and this training may have the added benefit of increasing hygienist self-efficacy to use the skills for other oral health-related behaviors among their patients. Clinical training in MI will be critical to not only understanding the key principles but also increasing clinician self-efficacy and effectiveness of implementation [52, 56]. Teens were mixed on whether they felt that it would be acceptable to receive this type of messaging in a dental setting. Some acknowledged the importance of hearing about meth use early (around 6th grade) before they are exposed in their peer groups, whereas others expressed concern that discussing meth and using “before and after” imagery during a dental visit could heighten some teens' existing fears of going to the dentist.

Teens and parents also noted that teens may feel that their dentist assumes or suspects they are using drugs if they bring up the topic of meth. A possible way to address this concern would be to communicate to teens that the intervention is standard and given to everyone within the dental practice to avoid teens feeling targeted and potentially defensive. This speaks to the need for the message and communication delivery method to be balanced, prevention-oriented (as not to have teens feel targeted), and acceptable to the target audience.

Teens felt that an image processing program that would morph their face and teeth to what it would look like after using meth could serve as an effective strategy if the images appeared realistic and were not viewed as a joke or game. Given the rapidity of dramatic changes in physical appearance and dental problems that can occur with meth use, the use of this type of program with the teen's own face could underscore the drastic cosmetic changes that can occur as a result of meth use. Restricting the use of the program to the dental setting would likely make the “before and after” images more impactful than if the program were useable by the broader public, as that may lead to it being viewed more as amusement. This is particularly relevant because loss-frame communication in substance use prevention (i.e., messages that highlight the negative aspects of behavior), such as physical changes associated with meth use, has been shown to be most effective when the quality of the message is perceived to be high [57].

The issue of intervention dose (i.e., how much the teen receives) was raised as a potential barrier to the efficacy of the intervention. Given that most teens only visit the dentist twice per year and some families cannot afford dental care, it is possible that it may not be strong enough as a stand-alone intervention. However, while this may not provide as strong of a dose as other settings (e.g., school curriculum), the most impactful prevention effects occur when teens' behaviors are targeted through multiple avenues, particularly at different levels of the social-ecological model [58–60]. Given that in 2019, 82.7% of youth 17 and under in Idaho had received preventive dental care in the past year [61], 85% of high school students saw a dentist in the past year [16], and dental professionals are considered credible messengers, adding an innovative setting to meth prevention will stand out and provide another layer of protection. One dentist articulated this point by saying: *“I think the success would be, not necessarily me teaching but me getting a discussion going at home. Hey, there is something that is not just talked about once every six months at the dental office. But it's talked about periodically, on a regular basis at home.” (Dentist: Private practice*)

There are many reasons that adolescents may engage in risky behaviors. In this context, two important reasons are as follows: first, they may feel that they are not vulnerable to particular harm associated with a behavior; second, they may not be aware of the consequences of that harm and not perceive their actions as unsafe. Framed in the context of the SCT, we have conceptualized a meth use preventive intervention to be delivered by dental hygienists, whereby teens will be shown an image of their face and teeth currently and then a simulation of what they might look like over a few time points if they were to use meth. Research has shown that fear appeal messages are often ineffective when used alone [62]. The EPPM, which has been empirically validated around numerous health topics, posits that a health message that utilizes fear is most effective when the recipients' perceived level of self-efficacy to address the fear is greater than that of the perceived threat [40, 63]. Because of this, the image processing component would be followed by a focus on meth-specific expectations and beliefs, self-regulation, and efficacy-building. The goal of this intervention would be to increase teens' perceived threat level and response self-efficacy level to a point that they can engage in refusal skills and proactively make decisions about choosing not to engage with peers who use or plan to use meth. Based on our research, this type of intervention should be limited to professional settings for it to be taken seriously and be maximally impactful.

### 4.1. Limitations and Strengths

There are a few notable limitations to this research. First, the interviews were exclusively conducted in North Idaho and, as such, the findings may not be generalizable to other parts of the state or country. Additionally, the interviews were conducted with a relatively small number of participants, all of whom were white and had preexisting knowledge of the extent and impact of meth use in the region, which also may indicate that the findings may not be broadly generalizable. However, the small group sizes facilitated conversation, which strengthened the findings to develop a communication-based prevention intervention. It is important to note that there was a time lapse between data collection and reporting of results because one of the investigators relocated across the country just after the data collection period thus stalling the analysis, and the authors have worked on presenting and publishing different aspects of this research [28]. In a sequential way, this publication is the last of those efforts. We feel that a significant strength of this study is the multiple perspectives on message delivery in the context of the dental setting.

## 5. Conclusions

To our knowledge, this research is the first of its kind to assess the feasibility and acceptability of delivering an innovative meth prevention intervention based on health communication and “before and after” imagery in the dental setting. While potential barriers were raised by teens and parents, this type of intervention was generally considered to be suitable for the setting and messenger, broadly appropriate in its content and messages for a teen audience with accompanying parental involvement, and to be potentially effective at preventing meth among teens. Practitioners in public health dental clinics found the intervention to be more acceptable and relevant to their daily patient care than did cosmetic and pediatric dentists who perceive their practices to be less impacted by meth use in their community, and as such, it will likely make sense to implement the intervention primarily in public health dental clinics as a first effort. Future qualitative research on meth prevention should consider how to include peers in interventions such as these. The findings provided important insight and considerations as to how to proceed with intervention development and implementation.

## Data Availability

The data that support the findings of this study may be available on request from the corresponding author. The data are not publicly available due to privacy or ethical restrictions.
